# Cell Structure of the Preoral Mycangia of *Xyleborus* (Coleoptera: Curculiondiae) Ambrosia Beetles

**DOI:** 10.3390/insects16060644

**Published:** 2025-06-19

**Authors:** Ross A. Joseph, Esther Tirmizi, Abolfazl Masoudi, Nemat O. Keyhani

**Affiliations:** Department of Biological Sciences, College of Liberal Arts and Sciences, University of Illinois at Chicago, Chicago, IL 60607, USA; rossj@uic.edu (R.A.J.); etirm2@uic.edu (E.T.); amasou7@uic.edu (A.M.)

**Keywords:** mycangia, cell structure, fungal mutualism, symbioses, fungal–insect interactions, ambrosia beetles, *Xyleborus affinis*, *Harringtonia lauricola*

## Abstract

Ambrosia beetles have evolved specialized structures termed “mycangia”, which house and transport symbiotic microbes. Microbial partners include at least one obligate mutualistic filamentous fungus used as food for larvae and adults, and potentially secondary filamentous fungi, yeasts, and bacteria. Beetles in the genus *Xyleborus* possess paired pre-oral mycangial structures located within the head on either side of the mouth parts. Mycangia develop in pupae, with newly emerged adults acquiring partners from the environment. However, information concerning the cellular structure and function of *Xyleborus* mycangia remains limited. We show that in *X. affinis*, mycangia are lined with a layer of striated dense material, enclosing layers of insect epithelial cells, with diverse spine-like structures. Larger (5–10 μm) projections were concentrated within and near the entrance of mycangia, with smaller filaments (4–8 μm) within the mycangia itself. Rows of “eyelash” structures lined the inside of mycangia, with fungal cells free-floating or in close association with these projections. Serial sections revealed mandibular articulations, and mandibular, pharyngeal, and labial muscles, along with the mycangial entry/exit channel. Sheets of comb-like spines at the mycangial entrance and opposite the mycangia attached to the roof of the labrum or epipharynx may serve as an interlocking mechanism for opening/closing the mycangia and guiding fungal cells into entry/exit channels. Additionally, mandibular fibra (muscle tissue) potentially enervating and affecting the mechanism of mycangial functioning were noted. These data add crucial mechanistic detail to the model of pre-oral mycangia in *Xyleborus* beetles, their cellular structures, and how they house and dispense microbial symbionts.

## Simple Summary

Fungal farming insects, which include certain species of ants, termites, beetles, and others, have independently evolved the unique ability to grow their own food. Ambrosia beetles represent one such group of insects, which farm their fungal partners in galleries constructed inside trees. The beetles maintain their galleries and tend their fungal “crops” as the sole source of food for larvae and adults. This association is so important to the beetles that they have evolved structures, termed mycangia, on their bodies used to house a small amount of the fungus to use as “seeds” for maintaining and/or initiating new fungal gardens. Here, we characterize the morphology and cellular structures that comprise the mycangia of *Xyleborus affinis* ambrosia beetles. We show that the mycangia are pouch-like, include specialized cellular layers that comprise their boundaries, and contain a series of projections, that include long, comb-like spines that may function as gates into and out of the mycangia. Muscle tissues localized near the mycangia may explain how the structure is manipulated for fungal entry and exit. Our data provide a model for the dynamic nature and functioning of this unique organ.

## 1. Introduction

The term “ambrosia beetle” describes a convergent lifestyle rather than a phylogenetic designation, where disparate beetle lineages have “domesticated” equally diverse genealogies of partner fungi [1,2,3]. Ranging from less than a millimeter to several millimeters in length, these small beetles bore into the sapwood of trees where they excavate galleries to rear their brood, with different species showing varying degrees of sociality [1,4]. Invasion of ambrosia beetles into new habitats, facilitated in part by transport of trees and lumber, can have profound effects on indigenous ecosystems, not only in terms of vectoring plant pathogens, but also on wood decay and turnover [5,6,7,8,9,10]. The beetles do not feed directly on tree components but rather cultivate their fungal partners along verdant walls of their galleries, often producing swollen, tender conidiospore-like cells upon which larvae and adults feed [11,12,13,14,15]. For ambrosia beetles, at least one fungal partner has become obligate. Via the process of this co-adaptation and evolution, likely over a span of tens to several hundred million years in some instances, the beetles have evolved specialized structures on or in their bodies, termed mycangia, to house and transport their fungal partner(s) [16,17,18,19]. Due to the polyphyletic nature of this adaptation, varied ambrosia beetle lineages have evolved completely different mycangia. Some mycangia are dorsal/pronotal pits and/or consist of elytral notches, whereas others include prothoratic tubes, mesonotal pouches, setose patches, prothoractic cavities, and preoral pockets/organs, the latter within the head of the beetle, around the mouthparts/mandibles [20].

Pre-oral mycangia are typically found in pairs inside the head, flanking the mouthparts and near the alimentary canal. From current descriptions, most ambrosia beetles that contain pre-oral mycangia belong to the subfamily Scolytinae, and are found in the Ipini, Xyleborini, and Xyloterini tribes, although, as mentioned, different genera within these tribes may have divergent mycangial structures, and examples of pre-oral mycangia can be found beyond these beetles [20]. Overall, structural and functional aspects of most mycangia remain significantly understudied; however, important insights have been gained in several exemplars. The pleural cavities found in adult *Trypodendron* (Xyloterini) are lined with gland cells that are activated when partner fungi enter, resulting in a milieu that favors selective propagation of the symbiont [21]. When the beetle, carrying its fungal inoculum, reaches a host tree, fungal cells are released during colony excavation, and the mycangia (gland cells) begin to deteriorate. As can be expected, the structure’s function in other types of mycangia can be radically different.

Sectioning and histological staining of pre-oral mycangia in *Euwallacea*, *Ambrosiodmus*, *Premnobius*, and *Xyleborus* beetles [17,22], has helped define ultrastructural aspects of these mycangia, revealing their shape, size, anatomical location, and connection to the foregut or pre-oral cavity. Non-invasive, micro-computed tomography (μCT) has also been used to visualize the pronotal (*Euplatypus*, *Scolytoplatypus*), mesonotal (*Xylosandrus*), and oral (*Ambrosiophilus*, *Euwallacea*) mycangia of a variety of beetles [17]. The dual pre-oral mycangia of *Euwallacea* beetles have been visualized by μCT across development and between sexes [23,24]. These latter data have shown the potential presence of numerous pre-oral mycangia in *Euwallaceae* beetles (superior and inferior mycangia), their development during pupation, and that, as adults, these structures appear fully “inflated” in the absence of colonizing fungal partners, in contrast to the mesonotal mycangia of *Xylosandrus* beetles, which have been shown to inflate upon exposure to partner fungi and deflate following their expulsion.

*Xyleborus* beetle species include invasive members whose fungal partners are highly destructive fungal tree pathogens [2,25]. *Xyleborus glabratus* is one such invasive species in the United States, and its fungal symbiont, since named *Harringtonia lauricola*, is the causative agent of laurel wilt, a disease that has killed hundreds of millions of redbay (*Persea borbonia*), swamp bay (*Persea palustris*), and sassafras (*Sassafras albidum*) trees, and now threatens the avocado industry in the United States [26,27,28]. *H. lauricola* has now been isolated from environmental samples of indigenous *Xyleborus* beetles, including the sugarcane shot-hole borer, *X. affinis*, and the ability of these latter beetles to maintain *H. lauricola* as their major symbiont has been confirmed in the laboratory [26,29,30]. These beetles have dual pre-oral mycangia located within the head, just behind and beneath the mandibles [30]. *Xyleborus* beetles can harbor fungal species now separated into the *Raffaelea*, *Harringtonia*, and *Dryadomyces* genera (all previously characterized as *Raffaelea*), as well as limited colonization by *Neocosmospora* (previously *Fusarium*) [14,31,32]. Mycangial colonization of *X. affinis* has been probed using fungal strains transformed to express reporter (*eGFP* and *RFP*)-marker genes [30]. These data showed stability to starvation as well as dynamic turnover, and transmission electron microscopy (TEM) showed a dimorphic shift in the growth of the fungus in the mycangia. *X. affinis* has been shown to be a good host for *H. lauricola*, with the related beetle species, *X. ferrugineus,* colonized to a considerably lower extent, indicating variation in colonization capacity between even closely related beetle species.

Here, we focus on structural and cellular aspects of the *X. affinis* pre-oral mycangia, defining novel morphological features that may contribute to partner selection, entry/exit, and/or fungal cell retention and maintenance within the mycangia, enriching and adding crucial detail to our understanding of these highly evolved organs and the essential symbioses that they regulate. Our data indicate the potential for specific muscle fibers (termed mandibular fibra) and specialized comb-like spines that may act in concert with mandibular movements to aid in the functioning of these mycangia as symbiotic fungal transport organs. By increasing our understanding of the functional and ultrastructural aspects of pre-oral mycangia, this study expands knowledge of the evolution and adaptations involved in fungal-animal symbioses.

## 2. Materials and Methods

### 2.1. Insect Rearing, Fungal Strains, and Culture Conditions

*Xyleborus affinis* colonies were maintained under laboratory conditions, and aposymbiotic beetles were reared for colonization experiments as previously described [30]. Briefly, sawdust agar medium was made by mixing 60 g wood flour, 15 g coarse sweetgum sawdust, 20 g agar, 10 g sucrose, 5 g corn starch, 5 g casein, 5 g yeast extract, 1 g Wesson salt mixture, and 2.5 mL wheat germ oil into 500 mL of water. Following autoclaving and cooling, 350 mg streptomycin and 10 mg tetracycline were suspended in 5 mL 95% ethanol and added to the mixture. Roughly 15–20 mL of this mixture was added to 50 mL conical tubes, and the tubes were allowed to dry for 7–10 days in the dark with caps lightly fastened. Colonies were initiated by scratching the surface of the sawdust medium with a scalpel to create a rough surface and adding 3–15 female beetles and 1–3 male beetles to each tube. Tubes were maintained with caps loosely tightened for 25–30 days in the dark at 23–25 °C. After this incubation period, colony tubes were carefully dissected to remove adult beetles, pupae, and larvae, with adult beetles used to initiate new colonies. Pupae and larvae were separated, surface sterilized with 70% ethanol for two minutes and three washes with sterile distilled water for one minute each, and maintained on sterile, moistened filtered paper until adult beetles emerged to generate aposymbiotic beetles for colonization assays. Fungal strains of *Harringtonia lauricola*, including those transformed to express green- or red-fluorescent protein (GFP/RFP), have been previously described [33], and unless otherwise noted, were routinely grown on potato dextrose agar (PDA) for use.

### 2.2. Microscopy

To obtain microscope images of fungal cells within *X. affinis* mycangia, aposymbiotic beetles were first colonized using GFP- and RFP-expressing *H. lauricola* by placing the beetles in individual wells of 96-well plates pre-inoculated with the fluorescent fungal strains and allowing them to feed on fungal conidia for as little as 1 h to as long as 7 days. Following this feeding period, whole beetles were embedded in Optimal Cutting Temperature (OCT) mounting medium (Sakura Finetek USA, Torrance, CA, USA) and frozen in a bath of isopentane cooled in liquid nitrogen. Frozen blocks were then sectioned using a Leica 3050S cryostat (Leica, Wetzlar, Germany) to a thickness of 5–20 μm. Sections were collected directly onto microscope slides, followed by fixation in 4% paraformaldehyde (PFA) for 20 min and three washes in sterile distilled water for three minutes each to remove PFA and OCT. Slides were then dried and mounted in Vectashield hardset mounting medium containing DAPI and phalloidin (Vector Laboratories, Plain City, OH, USA) for fluorescent staining of nuclei and actin filaments, respectively. Mounted sections were visualized using a Keyence BZX-800 fluorescence microscope (Osaka, Japan) at 40×, 60×, and 100× objective magnification using brightfield, TRITC, GFP, and DAPI fluorescent channels. For fluorescent images, Z stacks were collected over a range encompassing the signal and assembled into a full focus image in the Keyence BZX-800 Analyzer software V1.1.2.4, while for brightfield photos, single images were taken and overlayed with full-focus fluorescent images.

Transmission electron microscopy (TEM) images were obtained as previously described [30]. Briefly, aposymbiotic beetles fed on fluorescent fungal strains were mounted in OCT and frozen as above. Mounted beetles were sectioned to a thickness of 100 μm, and these sections were collected directly into wells of a 6-well plate containing 4% paraformaldehyde, 2.5% glutaraldehyde in a 0.1 M cacodylate buffer. Sections were fixed in this buffer by incubation at room temperature for 1 h at 50 rpm on a benchtop orbiter, followed by three washes in 0.1 M cacodylate buffer. After washing, sections were placed on a mesh grid and embedded in 3% low-gelling agarose for ease of handling. Embedded sections were then incubated in 2% osmium tetroxide in 0.1 M cacodylate buffer, followed by two washes with distilled water. Osmicated samples were then dehydrated in an ethanol series in 10% intervals from 25% to 100%, then to 100% anhydrous acetone. Dehydrated samples were embedded in Embed/Araldite resin and baked in an oven at 60 °C for 72 h to cure. Cured resin blocks were sectioned to a thickness of 120 nm and mounted onto slotted grids for TEM imaging. Mounted sections were stained with 2% uranyl acetate and lead citrate and imaged on an FEI Spirit 120 kV TEM. Measurements of anatomical structures from microscopy images were obtained using ImageJ software V1.53m (National Institutes of Health, Bethesda, MD, USA).

## 3. Results

### 3.1. Mandible, Muscle, and Cross-Section Analyses of the X. affinis Mycangia

**Figure 1:**
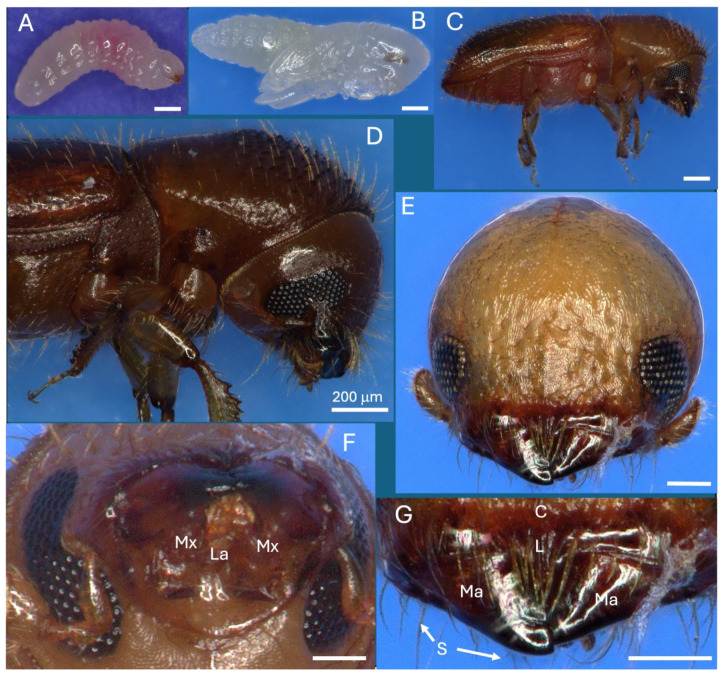
*X. affinis* life stages, head anatomy, and mouth parts. (**A**) Larva, (**B**) pupa, (**C**) adult, (**D**) adult sideview, (**E**) head, frontal view, (**F**) mandibles and maxillae, bottom view, (**G**) mandibles, top view. Scale bars: (**A**–**D**) = 200 mm, (**E**–**G**) = 100 mm. Abbreviations are as follows: Mx = maxillae, La = labium, Ma = mandibles, S = setae, L = labrum, C = clypeus.

*X. affinis* larvae, pupae, and adults are ~1.5, 2, and 2.1 mm in length (Figure 1A–C). Larvae pass through three instars, with adults emerging from pupae after 6–7 d. On adults, deeply emarginate compound eyes, located on either side of the mandibles, encompassed upper (~140 × 140 μm) and lower (~170 × 140 μm) patches of ommatidia containing the cornea, cones, and photoreceptor cells, with the small club-foot antennae characteristic of ambrosia beetles adjacent (Figure 1D,E). Mandibles (~150–200 μm) were located above the submentum and were multidentate and surrounded by dense setae (Figure 1E–G).

**Figure 2:**
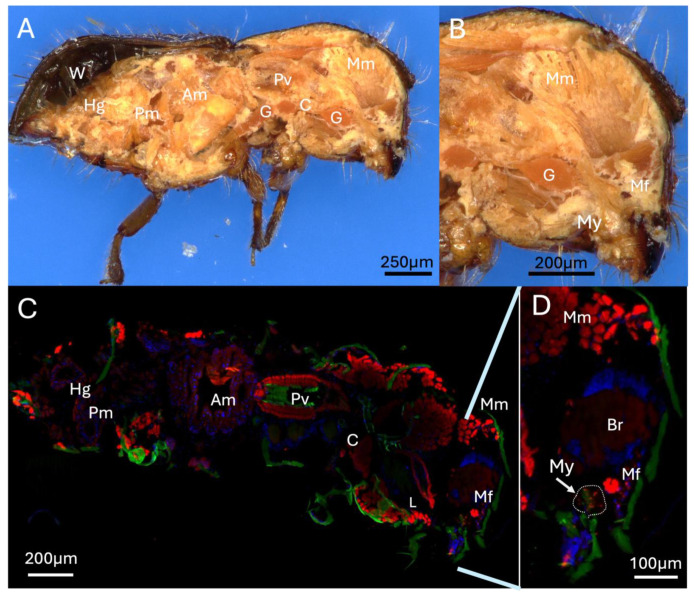
Longitudinal section of whole adult female beetle (**A**,**B**), examined via dissecting microscope, (**C**,**D**) fluorescence microscopy after DAPI nuclear (blue) and phalloidin (actin) (red) staining. Autofluorescence of insect tissues is shown in green. Abbreviations: W = wings, G = ganglia, Hg = hindgut, Pm = posterior midgut, Am = anterior midgut, Pv = proventriculus, C = crop, L = labial muscle, Mm = mandibular muscle, Mf = mandibular fibra, Br = brain, My = mycangia (circled).

Longitudinal cross-sectioning of whole adult female beetles revealed mycangia, gut tract, ganglia, and musculature, with significant mandibular muscles apparent extending from the head to the jaws (Figure 2A,B). A corresponding cross-section stained with DAPI and phalloidin and visualized via fluorescence microscopy revealed the major organs of the beetle, including the brain, hindgut, posterior-, and anterior-midguts, as well as proventriculus and crop (Figure 2C). Near/at the head region, the labial, pharyngeal, and mandibular muscles were also apparent (Figure 2C,D). In addition, strongly phalloidin-staining mandibular fibra, immediately adjacent (slightly above) the mycangia, were seen. As the beetle was fed a partner symbiotic fungus (*H. lauricola*) expressing RFP as detailed in the Methods Section, fungal cells within the mycangia could be detected via fluorescence microscopy. These cells were seen to localize exclusively to mycangia following colonization, indicating that host beetles can employ efficient mechanisms to guide partner fungal cells into mycangia and store them there without persistence of cells in surrounding insect anatomical structures.

**Figure 3:**
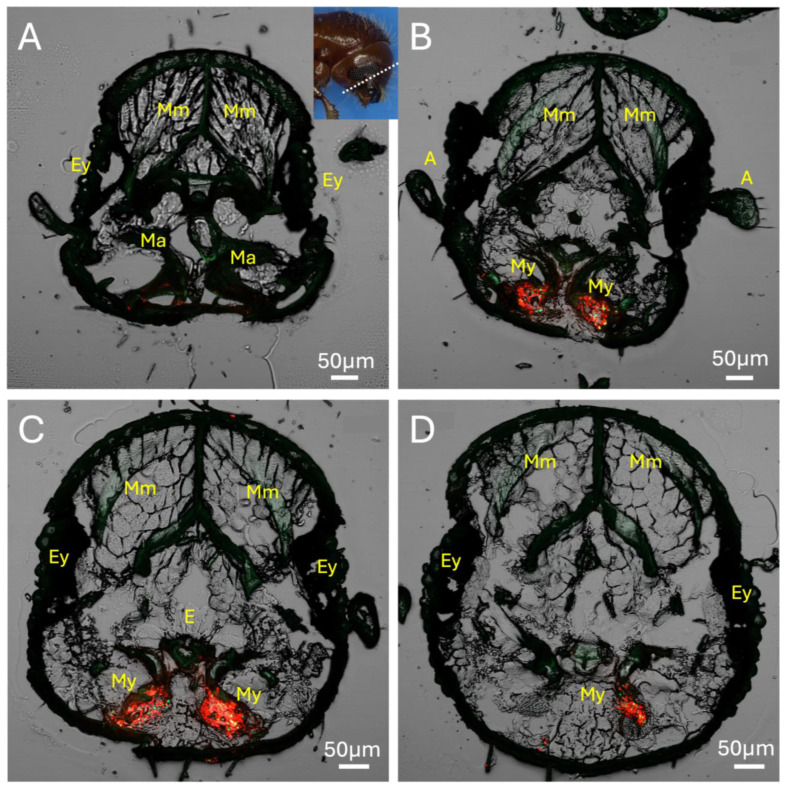
(**A**–**D**) Series of four serial sections through *X. affinis* head (location of sections shown by the dotted line within inset box of panel (**A**)) showing mandible articulations (Ma), mycangia (My), eyes (Ey), esophagus (E), antennae (A), mandibular muscles (Mm), pharyngeal muscles, and labial muscles. Beetle was colonized with GFP- (green fluorescence) and RFP-expressing (red fluorescence) *H. lauricola* cells, showing localization within mycangia. Autofluorescence of insect tissue is seen as faint green throughout the rest of the tissue sections. Note: Image in panel (**C**) republished with permission from [30].

Using red fluorescent protein (RFP) and green fluorescent protein (GFP)-expressing *H. lauricola* to mark the mycangia, a series of lateral cross-sections (20 μm, see inset in figure) of the mouthpart region revealed the overall outline and colonization of the two pre-oral mycangia seen in these beetles (Figure 3A–D). In the foremost section, mandibular articulations and muscles could be discerned, showing that these articulations sit directly in front of, and connect to, the mycangia via the chitinous lining of entry/exit canals (Figure 3A). Both mycangial pouches could be seen emerging in the next two sections, with the antennae, eyes, and esophagus also apparent (Figure 3B,C), with the fourth section likely exposing the outer posterior edge of one of the mycangial organs (Figure 3D). This posterior edge appeared as a thin translucent membranous layer textured by rows of small projections ordered neatly across the interior surface of this structure.

**Figure 4:**
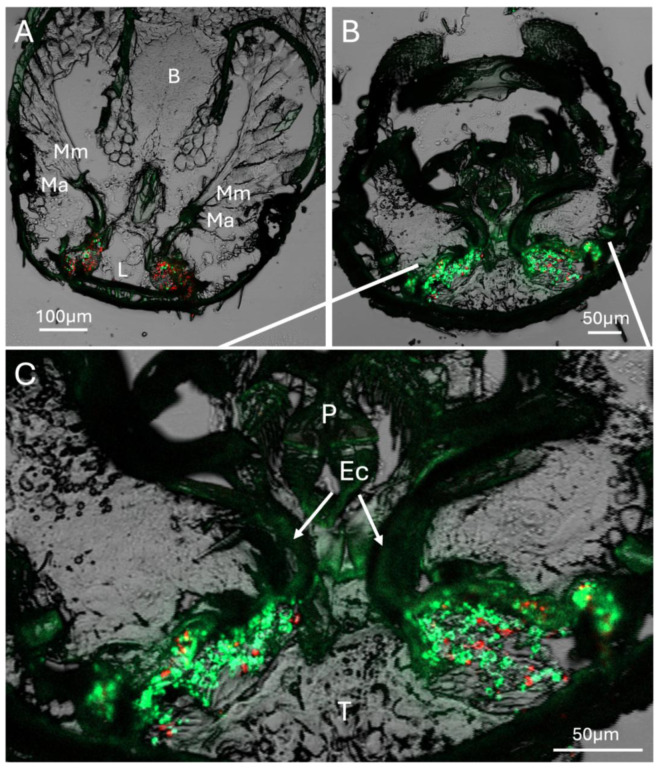
Sections showing head anatomy, including mycangia and mouthparts in more detail. (**A**) Section showing connection of mandibular muscles (Mm) to mandibular/mycangial articulation (Ma), brain (B), and labium (L). (**B**) Posterior section showing colonized mycangia with pinched ends. (**C**) Close-up detail of twin pre-oral mycangia. Ec = entry/exit channel, P = pharynx, T = tubular tissue region. Bright green and red (fluorescence) localized to mycangia represent GFP- and RFP-expressing *H. lauricola* cells, respectively. Faint green coloration throughout the rest of the tissue sections represents autofluorescence from insect tissue.

Further representative sections immediately within the mouthparts revealed connection of mandibular muscles to the mandibular/mycangial articulations, as well as outlines of entry/exit channels, which appeared as dense masses of melanized/sclerotized tissue with a furrow leading from the pre-oral cavity to the interior of the mycangia (Figure 4). The mycangial organs themselves were irregular in shape and were frequently pinched at the lateral end opposite the entry/exit canal. The furrowed entry/exit end was mainly noted in later (within the head) serial sections and appears to be a feature of the dorsal section of the mycangia, situated in the rear-most portion further from the mandibles. The entry/exit channels also appeared to be surrounded by a dense striated layer of tissue potentially functioning to contract/squeeze the mycangium, aiding in expulsion of fungal cells during inoculation of fungal gardens along gallery walls. As these beetles were fed a mixture of RFP- and GFP-expressing *H. lauricola* fungal symbiont partner cells, both green and red-fluorescent signals corresponding to the fungus could be discerned, showing robust colonization within the mycangial compartments. Almost all fungal cells noted in these, and subsequent analyses (and images) shown below, appeared to be single-celled blastospore/yeast-like cells apparently freely floating within the mycangia or in close association with spine-like projections along the inner mycangia lumen walls.

**Figure 5:**
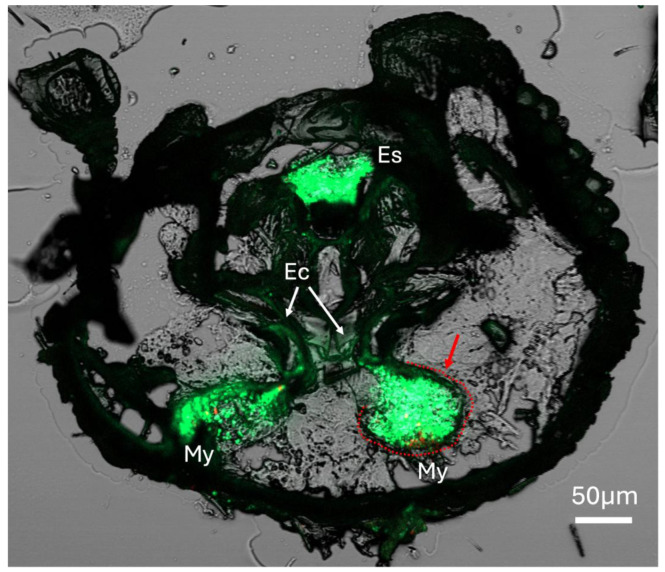
*X. affinis* beetle fed on fluorescent protein-expressing fungal cells, showing localization of fungal cells to mycangia and entry/exit canals, as well as fungal cells within the esophagus of the beetle. The red arrow and dashed line indicate a sclerotized structure outlining the mycangium. My = mycangium, Ec = entry/exit channel, Es = esophagus. Bright green and red fluorescent signals localized to mycangia, and esophagus represent GFP- and RFP-expressing *H. lauricola* cells, respectively. Faint green coloration throughout the rest of the tissue section represents autofluorescence of insect tissues.

In addition to the mycangia colonized by fungal cells, signals (of GFP-expressing fungal cells) could be seen within the entry/exit channels, as well as occasionally within the esophagus of the beetle, apparently denoting beetles that were frozen for cryosectioning while in the process of eating fungal cells (Figure 5). In these and previous sections, a clear sclerotized host structure could be discerned surrounding mycangial pouches, particularly in anterior sections closer to the mandibular articulations (Figure 5, marked with a dashed red line around one of the mycangial pouches).

### 3.2. Finer Details of the Surrounding External and Internal Structures of the Mycangia

**Figure 6:**
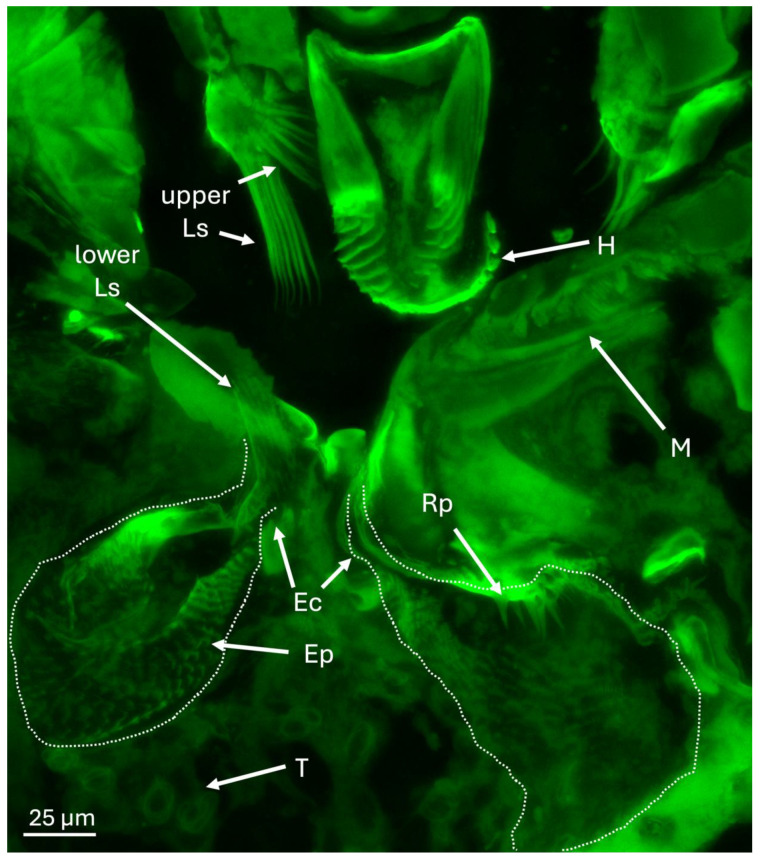
Epifluorescence image showing mouthparts and mycangia (dashed lines), including Ross projections (Rp), eyelash projections (Ep), entry/exit channels (Ec), hypopharynx (H), mandible (M), putative tracheoles (T), and upper and lower large comb-like spine structures (Ls) at the entrance to mycangia and opposite in pre-oral cavity. Green coloration is the result of autofluorescence of insect tissues.

Epi- (auto-) fluorescence microscopy of the area immediately surrounding the mycangia revealed the hypopharynx and mandibular articulations above and adjacent to the mycangial pouches (Figure 6). A series of spines and projections of various sizes within and into the mycangia, as well as near the entrance were noted. Upper and lower sets of large spines (Ls) at the entry/exit channels (Ec) were seen, which may guide fungal cells into mycangia and assist in their retention. Beneath and between mycangia, clusters of tubules were noted. In addition, Ross projections (Rp) along a portion inside the mycangia, as well as rows of “eyelash” projections (Ep) lining the inside, were seen.

**Figure 7:**
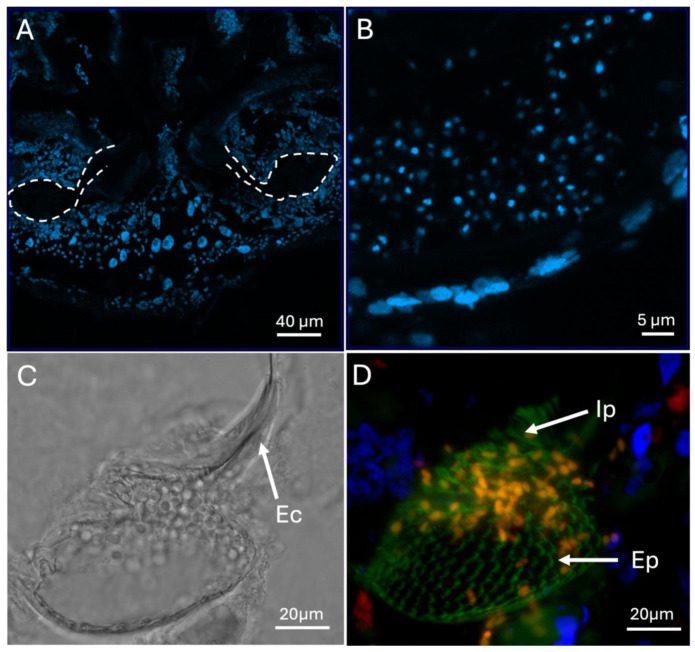
(**A**,**B**) DAPI-stained (blue) mycangial sections showing host cells surrounding the organ. Dashed lines in panel (**A**) outline the mycangia. (**C**) Bright field and (**D**) fluorescence images of mycangia and entry/exit channel (Ec), “eyelash” projections (Ep), and intermediate projections (Ip) lining the mycangia colored green by tissue autofluorescence. Fungal cells can be seen within the mycangia in the bright field and are colored orange in the fluorescent image due to multiple channel signals. Blue and red coloration in panel (**D**) results from nuclear DAPI-staining and actin phalloidin staining, respectively.

To further detail host cells forming a mycangial epithelium, sections were stained with DAPI. These images revealed that the mycangia is surrounded by several layers of cells (with small nuclei) that extended into the surrounding tissues. These cells became spaced further apart away from the mycangia and contained apparently larger nuclei. A tubular region between and below the mycangia, characterized by distinct large and spaced apart nuclei were noted (Figure 7A,B). Higher magnification brightfield and fluorescence images revealed the entry/exit channel, fungal cells within the mycangia, and rows of eyelash projections and intermediate projections lining the inner wall of the mycangia (Figure 7C,D).

**Figure 8:**
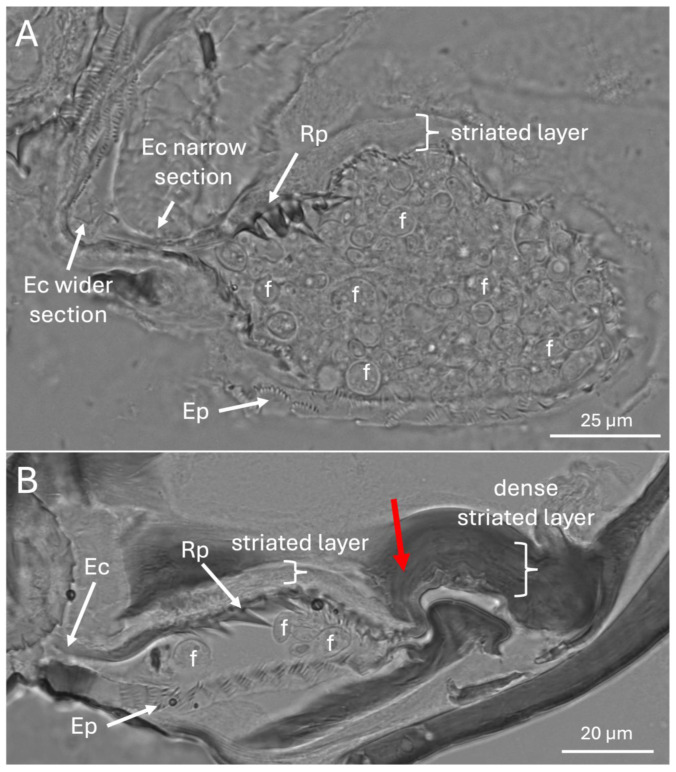
(**A**,**B**) Brightfield images showing Ross projections (Rp), eyelash projections (Ep), entry/exit channel (Ec), and fungal cells (f). Note mycangia in (**A**) is full of fungal cells, only some labeled with an “f”. In panel (**B**), the posterior invagination of the mycangia is marked with a red arrow.

The entry/exit channel appeared to have a wider (11–14 μm) initial region that narrowed to 1–5 μm as it reached the mycangia, with Ross projections apparent near the entryway. These larger projections gave way to smaller intermediate- and eyelash-projections which formed a surrounding layer within and facing into the mycangia (Figure 8A,B). Columns of striated tissues potentially corresponding to skeletal-like muscle were also seen near the beginning of the entry channel, and fungal cells were readily apparent within the mycangia.

**Figure 9:**
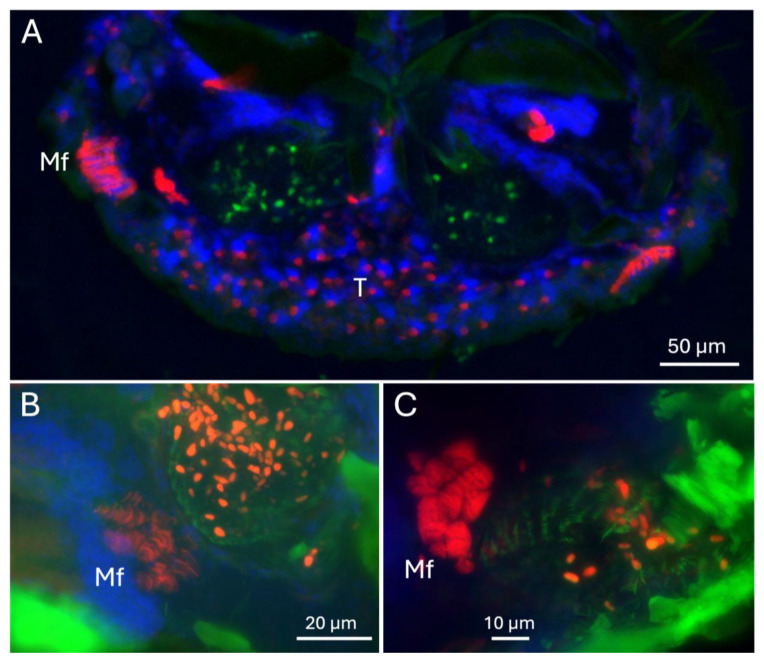
(**A**–**C**) Mf = mandibular fibra. T = tracheolar region showing large DAPI-staining nuclei (blue fluorescence) and numerous punctate actin phalloidin-staining (red fluorescence) points. Green punctate spots localized to the mycangia represent GFP-expressing *H. lauricola* cells and orange punctate spots represent RFP-expressing fungal cells (cells are orange due to multiple channel signals), while green fluorescence of surrounding insect tissue is the result of autofluorescence.

To further explore any muscle structures near the mycangia, sections were stained with phalloidin and counterstained with DAPI. Fluorescent microscopic images showed clear outlines of the mycangia (containing GFP-expressing fungal cells), surrounded by host cells with two sets of brightly phalloidin staining striated (muscle) structures on the sides of each mycangium. One set was found at the peripheral boundary of the mycangia and the other just above each mycangium separated by a layer of dense DAPI-staining host tissue (Figure 9A). These muscle structures on the peripheral boundaries (furthest from the entry/exit channels and esophagus) consist of 8–10 tightly bundled sets of fibers immediately adjacent to mycangia, while those above mycangia and bordering dense DAPI-staining tissues appear to consist of two–three brightly phalloidin-staining bundles. Striations in these muscle tissues ran parallel to or slightly angled from the walls of the mycangia. Higher magnification images, including epifluorescence images, revealed bundles (3–5) of columnar striated staining characteristic of skeletal muscle tissues at the periphery of the mycangia (Figure 9B,C).

**Figure 10:**
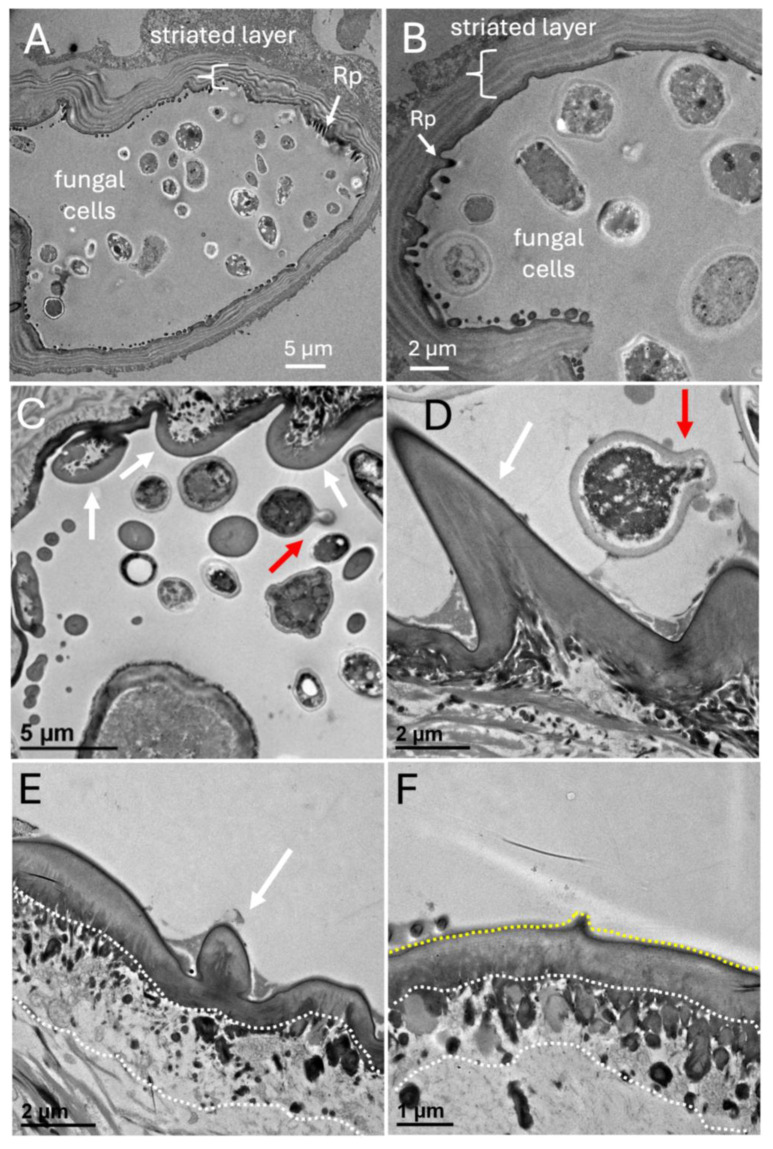
(**A**–**F**) TEM cross-sections of various projections/protrusion from inner layer of mycangial wall (white arrows) in *X. affinis* pre-oral mycangia, f = fungal cell, projection layer facing into mycangia (yellow dashed line), epithelial cell layer (underneath the projection layer, white dashed lines), and fungal cells (red arrows illustrate those fungal cells that appear to be budding). Note: Images in panels (**A**,**C**) are republished from [30] with permission; however, the presented images show a wider field of view.

**Figure 11:**
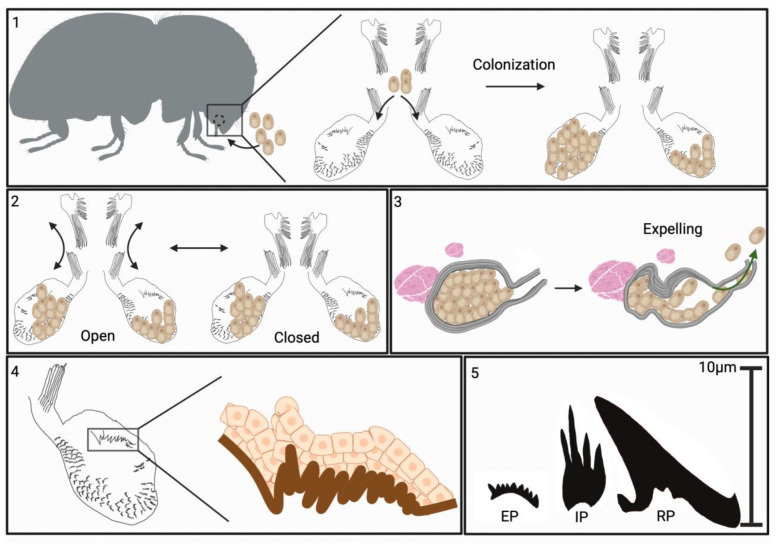
Summary model of *Xyleborus* mycangial features. Box 1: Mycangia structure and colonization: X. affinis mycangia are located in the heads of female beetles, just behind and below mandibles, and connected to the pre-oral cavity by narrow entry/exit channels. During feeding, fungal cells are guided into mycangia by long spines at the entrance of channels and opposite the furrows on the labrum. Inside the mycangia, the fungus grows as free-floating yeast-like cells. Box 2: Proposed mechanism of mycangial opening and closing: large spines situated at the roof of the pre-oral cavity, attached to the labrum, are flexed to open/close over the mycangial entrance. Association with projections within mycangia facilitates the retention of fungal cells. Box 3: Proposed mechanism of fungal cell expulsion from mycangia: Mycangia are surrounded by dense striated tissue hypothesized to be musculature. Bundles of muscle (fibra) were noted near the ends of mycangia opposite the entry/exit channel. Our data indicate that the mycangia can invaginate, often displaying a pinched end. We hypothesize that the musculature surrounding and adjacent to the mycangia can be flexed, squeezing these organs and facilitating expulsion of fungal cells during inoculation of gallery walls. Box 4: Cellular structures: mycangial wall tissue layers: innermost layer, directly exposed to the mycangia lumen, appears to be a hard sclerotized layer termed the “mycangial inner cuticular layer”. From this layer, different projections are noted: Ross projections; large spines present in a cluster near the entrance to the mycangia, tubules, composed of stacked oblong sections, intermediate projections; smaller, thinner, spines which appear to consist of two to three projections originating from a single base and clustering around Ross projections, and eyelash projections; small combs of spines which line the bottom and anterior edges of the interior layer. Surrounding (on the outside) the cuticular layer are layers of insect epithelial cells (DAPI staining and TEM imaging). Box 5: Projection types described from within mycangia and their relative sizes. EP = eyelash projections, IP = intermediate projections, RP = Ross projections.

TEM of cross-sections of the mycangia revealed a layer of striated material surrounding the mycangium (Figure 10A,B). Ross projections, as well as smaller projections directed into the mycangia which contained fungal cells, were also seen. The TEM images confirmed that most fungal cells within the mycangia displayed a free-floating yeast or blastospore-like growth morphology with variations in size and shape observed, as well as evidence of budding growth (red arrows, Figure 10C,D). Both short and longer Ross projections appeared to have a dense (in relation to the inside of the mycangia) layer, underneath of which was a layer of nucleated host cells. The cell layer in turn, gave way to surrounding rings of dense tissue (striations). Thus, the inner lining of the mycangia containing the short and longer Ross projections consisted of a layer (~1–1.5 μm) of dense material (mycangial cuticular layer) which was supported by a layer of mycangial epithelium (Figure 10E,F). The longer intermediate and/or Ross projections (e.g., extending ~6–10 μm in Figure 10B) appeared to consist of an extension of the mycangial dense layer, with nucleated host cells at its base. In order to integrate our findings, a summary model of the features of the *X. affinis* mycangia is given (Figure 11).

## 4. Discussion

As the ability to “carry” their fungal partners has evolved multiple times independently, the mycangia of ambrosia beetles represent different organs with varying locations, development, structure, and selection due to the different paths taken during their evolution [21,34]. Additionally, highly divergent forms of mycangia (also known as mycetangia) have been reported from other groups of insects, including species of wood wasp, stag beetle, and ship timber beetle [35,36,37]. Unlike the mycangia of ambrosia beetles, which vary widely in location on the insect body, are used to inoculate fungal gardens in galleries, and typically harbor filamentous fungi as their primary symbionts, the mycangia of these other groups of insects are often located close to the ovipositor of the insect, often harbor yeast symbionts, and are used to smear these fungal associates onto eggs after laying, facilitating the fitness of offspring via direct nutrition or breakdown of wood products, as well as the vertical transmission of the symbiont. The majority of Xyleborini genera, as well as some Xyloterini and Ipini species, have evolved a set of two pre-oral mycangia near the esophagus just behind the mandibles and connected to the alimentary canal via either the pre-oral cavity or the pharynx.

Traditional paraffin sectioning, laser ablation tomography (LATscan), and micro-computed tomography (μCT) have been employed to examine oral, pronotal, and mesonotal mycangia in a range of ambrosia beetles [17,20,21,23,24]. The mycangia of *Ambrosiodmus*, *Xylosandrus*, and *Scolytoplatypus* were characterized as being formed by an outer membrane containing the symbiotic fungus, with no glands or secretory cells visible. The mesonotal mycangia of *Xylosandrus compactus* and *X. discolor* have been shown to lie between the pronotum and scutellum, with a single bundle (pronoto-occipitalis) muscle. However, the more recent finer (e.g., via μCT) resolution imaging has shown that in *X. amputatus,* this muscle consists of two bundles, each of which splits into four sub-bundles as they cross, with each sub-bundle crossing each other, connecting from the right to the left sides of the scutellum [17]. Additional muscle bundles were found connected to the pronotum posterior. In conjunction with other muscle fibers, longitudinal contraction might constrict these types of mycangia, with deformation of muscle sets posited to occur during gallery excavation [16,17].

A μCT morphological analysis of female *Euwallacea validus* oral paired mycangia (partner fungus, *Fusarium oligoseptatum*) across larval, pupal, and adult stages revealed mycangial organ development during the late pupal stage, with the identification of two sets of mycangia, that could potentially be physically or functionally linked; one termed the superior medial mycangia (consisting of two pouches, located in front of the esophagus and behind the mandible), and the other the inferior, lateral mycangia (also consisting of two pouches, either side of head, behind eyes) [23]. In addition, the resolution of the method allowed for the putative identification of a rudimentary or proto-medial mycangium in males, previously unknown in male Xyleborini.

Confirming previous reports, our data show that the overall structure of the *X. affinis* preoral mycangia consists of two irregular-shaped pouch-like organs directly behind and beneath the mandibles and connected to the esophagus via the pre-oral cavity. These pouches appeared to be connected to the mandibular articulations via the entry/exit channel at their frontal end, potentially allowing for their articulation in conjunction with mandibular movements and by mandibular and other sets of muscles. We further elaborate on the entry/exit channels of each mycangium, which appear as furrows with a wider initial segment that narrowed significantly into the mycangium, and were surrounded by dense tissue matter, potentially muscular in nature, but which did not stain with phalloidin. At the mycangial entrance, sets (upper and lower) of long, comb-like spines extending from the labrum or epipharynx “downwards” and from the entrance of the entry/exit channel “upwards” were identified. We hypothesize that these structures, if enervated by muscle tissues and linked to movements of the mandibles, labrum, or epiharynx, could act as gates or portcullises to the mycangia, functioning to retain fungal cells during dispersal. Though largely reliant on morphological information from this study, future experiments, employing live or biomechanical techniques to examine these structures in motion, may add additional evidence and credence to these hypotheses.

The mycangial envelope itself appeared to have three discernible layers (from the inside out): (i) a dense staining layer (0.5–2.5 mm) devoid of defined features, but which extended in places into the mycangial lumen, forming spines and longer Ross projections. (ii) This was followed by a middle layer of apparent epithelial-like (nucleated) cells (2–5 cells thick), which could also be seen at the base of the spines and projections. (iii) The outermost or peripheral section of the mycangia consisted of a striated layer (1–10 mm, 3–5 bands), which did not appear to be comprised nucleated cells or actin (i.e., did not stain with DAPI or phalloidin). In the posterior sections of mycangia, we consistently noted that this striated layer, and subsequently the mycangial pouch, appeared pinched in appearance, suggesting that this portion of these organs may be flexible, and potentially could be squeezed by surrounding musculature as a mechanism of expelling fungal cells from mycangia during gallery excavation and fungal garden inoculation.

Within or directed into the mycangia from the inner wall, a variety of previously described as well as unreported structures were noted. Larger spines or Ross projections, apparently concentrated in clumps, sometimes, but not always, near or just within the mycangial entrance, have been noted before. The mycangial lumen contained rows of ordered eyelash-like structures lining the inner mycangial wall (here termed eyelash projections). In addition, tubule-like structures consisting of stacked tubes leading to filaments, as well as structures typically with three–five filaments of intermediate size projecting from a diffuse base (intermediate projections), were characterized. Duct-like structures, spines, and hair-like protrusions have been noted for a variety of mycangia, most notably in *Ambrososiophilus atractus*, *Premnobius cavipennis*, *Ambrosiodmus lecontei*, and *E. validus* [22,38,39]. The functions of these enigmatic structures remain to be elucidated, with various hypotheses related to selection and maintenance, coupled with the potential for nutrient and/or signaling exchange, warranting further experimentation.

## 5. Conclusions

The current work, demonstrating the interior of pre-oral mycangia to contain layers of epithelial cells, indicates dynamic biological functioning beyond inert mechanical retention of fungal cells. Recently, pathways involved in insect tubulogenesis have been implicated in mycangial development [40]. In addition, new techniques for analysis of mycangial contents are allowing for better characterization of how mycangia are colonized by partner fungi [41]. Our observations provide a detailed morphological characterization of the pre-oral mycangia of a *Xyleborus* ambrosia beetle at the ultrastructural scale. This has allowed for proposing a hypotheses regarding their function as symbiont housing, transporting, and dispensing organs. Specific mechanisms including surrounding muscle tissues for mediating entry and exit of fungal cells, host structures that may participate in fungal recruitment and retention (spines and projections), and host cells that may provide for nutritional exchange were characterized. Future studies aimed at testing the hypotheses developed in this manuscript may help to further shed light on the roles of the different anatomical structures described herein and their specific involvement in the overall functioning of this highly successful symbiotic system, including the flexibility/mobility of various structures and the identity of distinct cell types within the mycangial cell layers noted here. Of particular interest may be the identification of potential glandular cells which have not previously been reported within pre-oral mycangia, and to what extent the spines guarding the entrance to mycangia are able to flex or move to facilitate fungal cell retention. Such knowledge would greatly aid in the understanding of the ecology and evolution of fungal-animal symbioses, particularly in the Xyleborini and their fungal associates, a large and impactful group of organisms.

## Data Availability

The original contributions presented in this study are included in the article. Further inquiries can be directed to the corresponding author.
